# Advancing allergen characterization in Perilla seed: an oil body purification approach

**DOI:** 10.1186/s13223-026-01012-6

**Published:** 2026-01-30

**Authors:** Kyunguk Jeong, Eunjung Kim, Purevsan Gantulga, Se-Ah Jeon, Sooyoung Lee

**Affiliations:** Department of Pediatrics, Ajou University School of Medicine, 164 Worldcup-ro, Yeongtong-gu, Suwon, Gyeonggi-do 16499 Republic of Korea

**Keywords:** Allergen characterization, Immediate-type hypersensitivity, Oil body purification, Oleosin, Perilla seed, Seed allergies

## Abstract

Perilla seed (PS), a common food in East Asia, has emerged as a leading cause of seed-induced anaphylaxis in Korean children, yet its allergenic proteins remain unidentified. Oleosins, hydrophobic oil-body (OB)–associated storage proteins, are recognized allergens in peanuts, hazelnuts, and sesame, and preliminary evidence suggests a similar role in PS. However, the intrinsic challenges of extracting lipophilic proteins have hindered systematic investigation. This study aimed to purify PS OBs, isolate oleosin under optimized conditions, and characterize candidate major allergens. Commercial Korean PSs were homogenized, and OBs were purified via sequential flotation. After defatting, proteins were separated by SDS-PAGE and tested by immunoglobulin E (IgE) immunoblotting using sera from pediatric PS-allergic patients. Protein bands corresponding to IgE binding were excised for LC–MS/MS analysis, and peptide spectra were searched against an in-house database using MASCOT. SDS-PAGE revealed multiple protein bands between 12 and 15, 20, 30–35, and ~ 50 kDa. IgE binding was observed at 12, 14–15, 20, 33, and 50 kDa, with inter-individual variability. LC–MS/MS consistently identified PS oleosin and oleosin-like proteins in the 12–15 kDa and 33 kDa regions, with the 14–15 kDa band showing the strongest identification (Mascot score up to 190). In conclusion, oleosin at 14–15 kDa, along with a probable 33 kDa dimeric form, represents the predominant allergenic protein in PS. This study provides the first systematic molecular characterization of PS oleosins and establishes a methodological framework for studying hydrophobic seed allergens.

**Supplementary Information:**

The online version contains supplementary material available at 10.1186/s13223-026-01012-6.

## Background

Globally, seed consumption has increased, with a concomitant rise in reported seed allergies [1]. However, most studies have focused almost exclusively on sesame seed, with limited reports on sunflower, mustard, and flaxseed, and virtually no reports on perilla (*Perilla frutescens*) seed. Perilla seeds (PSs) are widely consumed in East Asia and have been reported as the most frequent cause of seed-induced anaphylaxis in Korean children [1–3]. Apart from a single case report describing two patients with PS-induced anaphylaxis, no systematic investigations were available until our group reported the clinical and immunological features of more than 20 pediatric patients with PS allergy in 2023 [4–7].

Seed storage proteins are generally regarded as major allergens in nuts and seeds; however, allergen research on seeds has been largely restricted to sesame [8]. PS allergens remain unidentified despite PS being a clinically important trigger for immediate-type hypersensitivity. Lipophilic proteins have been suggested as potential allergens, and oleosin—an oil body (OB)-associated protein—has been identified as an allergen in peanuts, hazelnuts, and sesame (Ses i 4 and Ses i 5) and may be linked to severe allergic reactions [9–11]. In our previous study, preliminary evidence suggested a role for oleosin as a major allergen in PS; however, the intrinsic features of oleosin, namely its strong hydrophobicity, susceptibility to contamination, and difficulties in extraction, have limited allergenicity studies of PS and other plant foods [7, 12, 13].

Therefore, this study performed independent purification of OBs from PS; enhanced the subsequent isolation and analysis of oleosin; and characterized candidate major allergens, with the aims of advancing technical approaches for oleosin extraction and defining its potential role as a major plant food allergen.

## Methods

Commercially available pure Korean PSs were homogenized, and OBs were purified through sequential flotation steps and adjusted to 100 mg lipid/mL [14]. After defatting, the proteins were separated by sodium dodecyl sulfate-polyacrylamide gel electrophoresis (SDS-PAGE) and tested against sera from pediatric patients with clinical PS allergy, under optimized immunoglobulin E (IgE) immunoblotting conditions. The detailed methods are provided in Additional file 1.

SDS-PAGE-separated protein bands of approximately 12, 14–15, 20, 33, and 50 kDa were excised for amino acid sequencing analysis. Gel pieces were washed in 25 mM ammonium bicarbonate/50% acetonitrile, dehydrated, rehydrated with trypsin, and incubated overnight at 37 °C. Peptides were extracted with 1% formic acid/50% acetonitrile, concentrated, and desalted using reversed-phase microcolumns. Liquid chromatography–tandem mass spectrometry (LC–MS/MS) analysis was performed using a nano ACQUITY UPLC coupled to an LTQ Orbitrap mass spectrometer (Thermo Electron, San Jose, CA, USA). The spectra were processed using SEQUEST (Thermo Quest) and searched against an in-house database using MASCOT (Matrix Science Ltd., London, UK). MASCOT scores of > 48 were considered reliable. Detailed chromatographic and MS conditions are provided in Additional file 1.

## Results

**Fig. 1:**
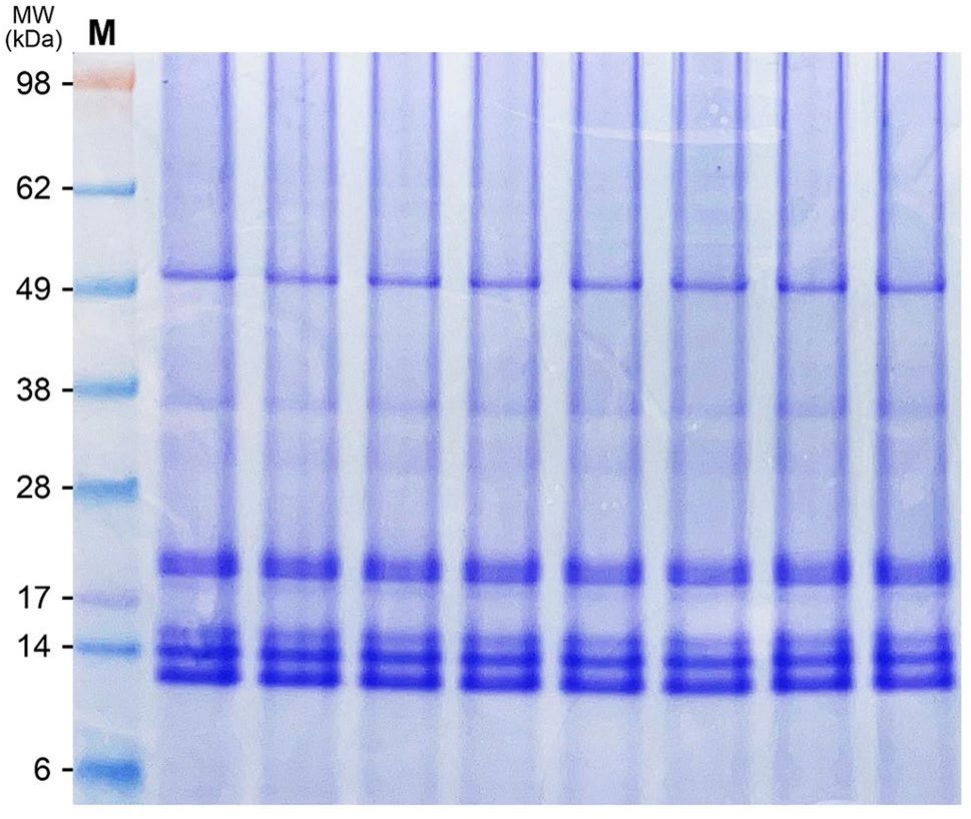
SDS-PAGE analysis of defatted oil body proteins from perilla seed, stained with Coomassie brilliant blue R-250

**Fig. 2:**
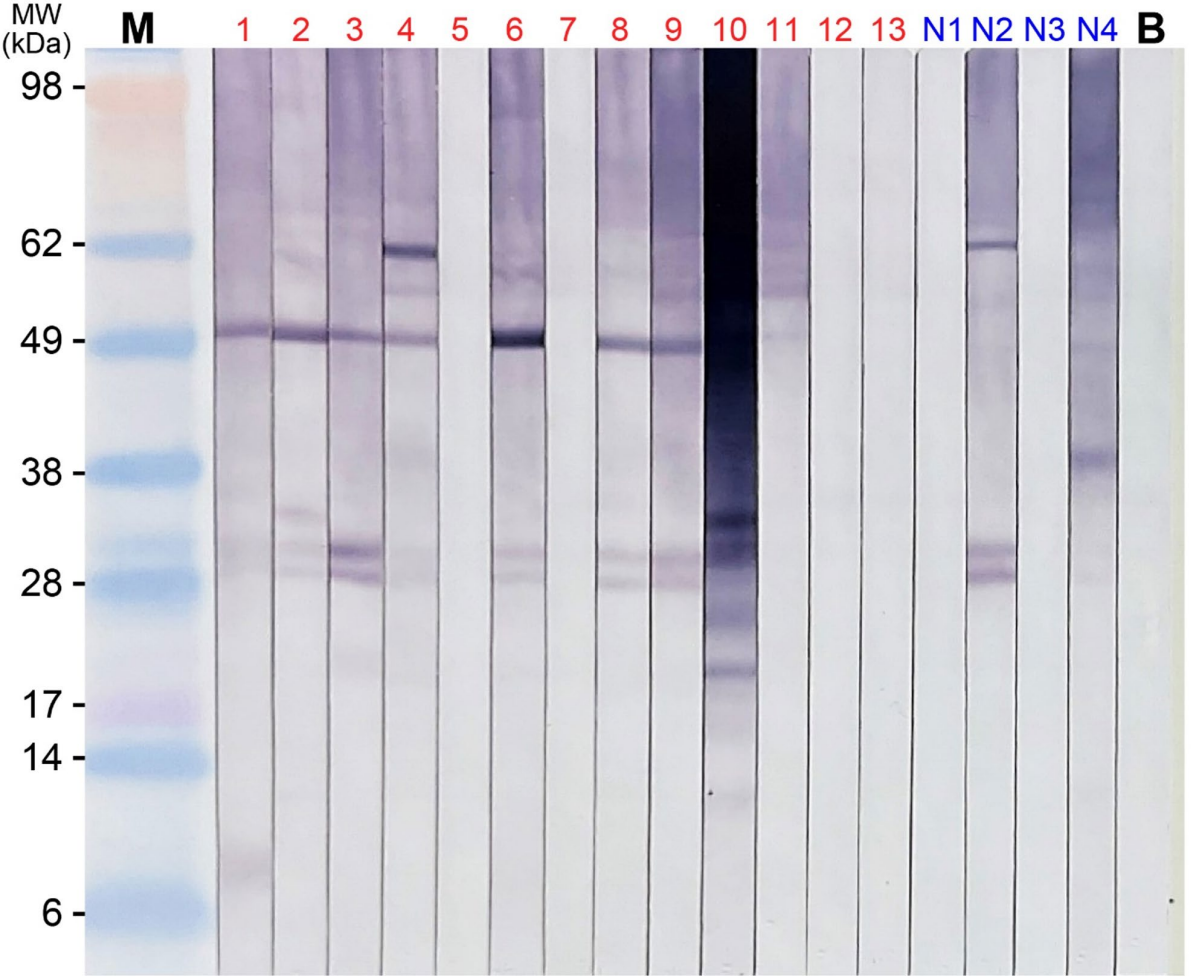
IgE-immunoblot analysis of defatted oil body proteins from perilla seed. Lanes 1–13 correspond to individual sera from pediatric patients with clinical perilla seed allergy (positive sera), lanes N1–N4 represent negative sera, and lane B is the blank control. Distinct IgE-binding signals are observed primarily in the 12, 14–15, 20, 33, and 50 kDa regions, varying across individual patients. IgE, immunoglobulin E

SDS-PAGE of the isolated OBs revealed multiple protein bands at 12–15, 20, 30–35, and ~ 50 kDa (Fig. 1). IgE-immunoblotting using sera from patients with clinical PS allergy showed binding at 12, 14–15, 20, 33, and 50 kDa; inter-individual variability was noted (Fig. 2). LC–MS/MS analysis identified multiple oleosin-related proteins across different molecular weight regions (Table 1). In the 14 kDa band, oleosin (*P. frutescens*) was the most prominent protein, with a Mascot score of 190 and 109 peptide matches, representing the strongest identification in the dataset. A 15 kDa oleosin-like protein 1 from perilla was also detected in the 14 kDa band, with a score of 59. In the 12 kDa band, a 15 kDa oleosin-like protein 1 was identified (score 65, 6 peptide matches). The 15 kDa band showed oleosin (*P. frutescens*) (score 127, 19 peptide matches), together with another oleosin-related hit from the same species (score 190). In the 33 kDa band, oleosin (*P. frutescens*) was strongly detected (score 127, 23 peptide matches), indicating that oleosin can appear at multiple molecular weights, possibly reflecting isoforms, processing variants, or oligomeric states. In contrast, no oleosin or oleosin-like proteins were identified in the 20 or 50 kDa bands, where other storage proteins or low-confidence hits were predominant. Taken together, these findings demonstrate that oleosin and oleosin-like proteins from *P. frutescens* are consistently and robustly identified in the 12–15 and 33 kDa regions, highlighting their predominance among proteins detected in the PS OB fractions.

**Table 1:** LC–MS/MS profiling of proteins isolated from Perilla seed oil bodies, with mascot scores > 48 considered significant

Sample	MASCOT Score	Protein name	Species	MW	Peptide matches
50 kDa	86	Unnamed protein product	*Arabis nemorensis*	51,380	6
	86	Hypothetical protein SELMODRAFT_116103	*Selaginella moellendorffii*	47,371	6
	73	Hypothetical protein Ccrd_021776	*Cynara cardunculus var. scolymus*	48,291	8
	73	Hypothetical protein C5167_031276	*Papaver somniferum*	23,444	8
	64	Hypothetical protein GOBAR_AA14636	*Gossypium barbadense*	38,934	6
	59	Hypothetical protein Saspl_052934	*Salvia splendens*	50,840	6
33 kDa	127	**oleosin**	***Perilla frutescens***	18,726	23
	71	40 S ribosomal S3-3 -like protein	*Gossypium arboreum*	26,470	4
	61	Hypothetical protein GOBAR_AA14636	*Gossypium barbadense*	38,934	10
	58	Oleosin	*Striga asiatica*	25,401	1
	50	Germinal-center associated nuclear protein	*Ananas comosus*	159,719	1
	50	Alpha-glucan water dikinase 2 isoform X1	*Abrus precatorius*	152,668	1
20 kDa	159	Hypothetical protein Saspl_052934	*Salvia splendens*	50,840	45
	140	Hypothetical protein Saspl_012488	*Salvia splendens*	51,266	45
	138	Hypothetical protein Saspl_051912	*Salvia splendens*	48,562	10
	127	Hypothetical protein Saspl_027449	*Salvia splendens*	56,005	41
	88	Hypothetical protein BC332_10107	*Capsicum chinense*	43,299	6
	80	Unnamed protein product	*Arabis nemorensis*	18,043	3
	68	**Legumin-like protein**	***Perilla frutescens***	52,987	6
	65	60 S ribosomal protein L12	*Tetrabaena socialis*	17,831	2
	61	Probable LRR receptor-like serine/threonine-protein kinase IRK	*Phoenix dactylifera*	105,140	11
	60	RecName: Full = Translationally-controlled tumor protein homolog; Short = TCTP; AltName: Full = CmTCTP	*Cucurbita maxima*	19,146	1
	60	Hypothetical protein TEA_002880	*Camellia sinensis var. sinensis*	14,386	1
	59	11 S globulin	*Pistacia vera*	53,516	8
	55	11 S seed storage globulin	*Chenopodium quinoa*	54,007	4
	55	Manganese superoxide dismutase, partial	*Litchi chinensis*	24,475	1
	55	Arylacetamide deacetylase	*Handroanthus impetiginosus*	35,744	5
	52	Hypothetical protein EJD97_013512	*Solanum chilense*	53,762	2
	51	PREDICTED: TGACG-sequence-specific DNA-binding protein TGA-2.1-like	*Gossypium arboreum*	28,791	3
	51	Hypothetical protein DCAR_005738	*Daucus carota subsp. sativus*	108,129	9
	51	SET domain-containing protein	*Cephalotus follicularis*	105,752	9
15 kDa	127	**Oleosin**	***Perilla frutescens***	18,726	19
	50	Unnamed protein product	*Ananas comosus var. bracteatus*	38,234	1
14 kDa	190	**Oleosin**	***Perilla frutescens***	18,726	109
	63	RecName: Full = Histone H2A	*Euphorbia esula*	16,048	2
	63	Unnamed protein product	*Arabis nemorensis*	14,019	2
	59	**15kD oleosin-like protein 1**	***Perilla frutescens***	15,110	3
12 kDa	65	**15kD oleosin-like protein 1**	***Perilla frutescens***	15,110	6

## Discussion

In this study, we identified PS oleosin at 14–15 kDa as the principal candidate allergen in *P. frutescens*, with an additional 33 kDa oleosin band likely representing the dimeric form. These findings parallel those of oleosins previously recognized as allergens in other plant foods, including peanuts (Ara h 10, Ara h 11, Ara h 14, Ara h 15), hazelnuts (Cor a 12, Cor a 13, Cor a 15), and sesame (Ses i 4, Ses i 5), all within the 14–18 kDa range [10, 11, 15]. These findings are supported by emerging evidence suggesting that oleosins from flaxseed, soy, sunflower, and walnut are potential allergens, although they have not yet been formally registered [16, 17]. Compared with our previous study using conventional plant protein extraction (defatting followed by Phosphate Buffered Saline extraction and dialysis), the present work is distinguished by the use of OB purification tailored to the hydrophobic nature of oleosin, representing an important methodological advancement. Nevertheless, OB purification remains technically challenging and lacks standardized protocols, with risks of oleosin loss or denaturation during organic solvent treatment, as well as oligomer formation due to the self-binding of hydrophobic domains following lipid removal [9, 16]. Further optimization may include testing diverse salt conditions (e.g., NaCl, KCl, NaHCO₃, MgCl₂, CaCl₂), refining acetone washing steps to minimize protein loss (e.g., a single cold wash with immediate drying), and applying post-isolation anti-aggregation strategies using low concentrations of non-ionic detergents.

Although our previous study demonstrated that extracted PS proteins were capable of triggering mediator release from effector cells, SPT and/or basophil activation testing using the OB-purified protein extract could not be included in the present dataset, representing a remaining limitation of this work. Future research should also incorporate SPT and/or basophil activation test using the OB-purified protein to validate its functional allergenicity, and should also focus on elucidating the primary and secondary structures of PS oleosins, mapping IgE-binding epitopes, and exploring cross-reactivity with oleosins from other plant foods through sequence homology and immunological assays.

## Supplementary Information

Below is the link to the electronic supplementary material.

Supplementary Material 1

## Data Availability

The datasets used and/or analysed during the current study are available from the corresponding author on reasonable request.

## Abbreviations

IgE: immunoglobulin E
LC-MS/MS: Liquid Chromatography-Tandem Mass Spectrometry
OB: oil body
PS: perilla seed
SDS-PAGE: sodium dodecyl sulfate–polyacrylamide gel electrophoresis
