# Supplementary material for: Advancing allergen characterization in Perilla seed: an oil body purification approach
Source: Allergy Asthma Clin Immunol. 2026 Jan 30;22:6. doi: 10.1186/s13223-026-01012-6 (PMC12882140; doi:10.1186/s13223-026-01012-6)
Supplement: Supplementary file 1 — Supplementary Material 1 [file 13223_2026_1012_MOESM1_ESM.docx]

Additional File 1. Stepwise protocols and reagent formulations for perilla seed oil body extraction and LC–MS/MS–based protein analysis

| OB purification from PS | PSs (50 g) were homogenized for 5 min in chilled grinding buffer (GB: 1 mM EDTA, 10 mM KCl, 1 mM MgCl₂, 2 mM DTT, 0.15 M Tricine, 0.6 M sucrose, pH 7.5 adjusted with KOH) at a ratio of 1:2 (w/v) using a blender. The homogenate was filtered through gauze and mixed 1:1 (v/v) with flotation buffer (FB: GB containing 0.4 M sucrose). After centrifugation at 10,000 × g for 30 min at 4 °C, the floating oil body layer was collected, resuspended in detergent washing solution (DWsol: GB containing 0.2 M sucrose, 0.1% Tween-20, 75mM Tricine, pH 7.5), and layered with 150 mM Tricine (pH 7.5) at a 1:1 ratio prior to centrifugation. The recovered floating layer was then resuspended in resuspension buffer (RB: GB containing 2 M NaCl) and subjected to centrifugation after layered on top 1:1 (v/v) with FB. The floating fraction was resuspended in 8 M urea, gently mixed at room temperature (60 rpm, 10 min), and centrifuged again after layered 1:1 (v/v) with 150 mM Tricine (pH 7.5). The floating layer was re-suspended in GB, layered on top 1:1 (v/v) with FB, and centrifuged. The final floating layer was re-suspended in GB and adjusted to a lipid concentration of approximately 100 mg/mL. |
| --- | --- |
| Delipidation of OBs | Isolated oleosomes were placed in 50 mL centrifuge tubes and mixed with 2 volumes of chilled diethyl ether to disrupt the oil body structure and release neutral lipids. After centrifugation (13,600 × g, 4 min, 4 °C), the aqueous layer treated with 2 volumes of chloroform-methanol (2:1 v/v) and centrifuged. The upper methanol and lower chloroform layers were removed. Then the remaining interfacial materials washed with 3 times with 1 volume of distilled water and 3 volume of chloroform-methanol (2:1 v/v) and centrifuged. The resulting pellet was dried and stored at –80 °C until use. |
| In-gel digestion and peptide extraction | Protein bands separated by SDS-PAGE were excised and subjected to in-gel trypsin digestion. Excised gel pieces were washed for 1 h at room temperature in 25 mM ammonium bicarbonate (pH 7.8) with 50% acetonitrile (ACN), dehydrated in a SpeedVac™ concentrator (Thermo Scientific), rehydrated in sequencing-grade trypsin (20 ng; Promega, Madison, WI, USA), and incubated overnight at 37 °C. Tryptic peptides were extracted with 1% formic acid (FA)/50% ACN, concentrated by vacuum centrifugation, and desalted using reversed-phase microcolumns. |
| Protein identification by LC–MS/MS | LC–MS/MS was performed using a nano ACQUITY UPLC coupled to an LTQ Orbitrap mass spectrometer (Thermo Electron, San Jose, CA) with a BEH C18 column (1.7 μm, 100 μm × 100 mm; Waters, Milford, MA, USA). The mobile phases were 0.1% FA in water (A) and 0.1% FA in ACN (B), and peptides were separated with a linear gradient from 10% to 40% B over 21 min, 40% to 95% B over 7 min, followed by re-equilibration to 10% B over 10 min, at a flow rate of 0.5 μL/min. Data-dependent acquisition included full MS scans (m/z 300–2000) followed by MS/MS. The ion transfer tube was maintained at 275 °C, with a spray voltage of 2.0 kV and normalized collision energy of 35%. MS/MS spectra were processed using SEQUEST (Thermo Quest, San Jose, CA, USA) and searched against an in-house database with MASCOT (Matrix Science, London, UK). Search parameters included carbamidomethylation (C) as a fixed modification and deamidation (NQ), oxidation (M), and phosphorylation (S/T/Y) as variable modifications. Peptide mass tolerance was set at 2 Da, MS/MS tolerance at 1 Da, with up to two missed cleavages and charge states of +2 and +3 allowed. |
